# Diaqua­bis­[5-(1-oxidopyridin-1-ium-2-yl)-1,2,3,4-tetrazolido]manganese(II) di­hydrate

**DOI:** 10.1107/S1600536811001620

**Published:** 2011-01-15

**Authors:** Feng Gao, Chang-Sheng Yao, Zai-Sheng Lu, Yan-Hui Shi

**Affiliations:** a School of Chemistry and Chemical Engineering, Xuzhou Normal University, Xuzhou 221116 Jiangsu, People’s Republic of China

## Abstract

In the title compound, [Mn(C_6_H_4_N_5_O)_2_(H_2_O)_2_]·2H_2_O, the Mn^II^ ion is situated on an inversion centre and is coordinated by the O and N atoms of two bis-chelating 5-(2-pyridyl-1-oxide)tetra­zolate ligands and two O atoms of two water mol­ecules in a distorted octa­hedral geometry. All the water H atoms are involved in O—H⋯N and O—H⋯O hydrogen bonds with uncoordinated water O atoms and tetra­zole N atoms, which link the mol­ecules into a three-dimensional network.

## Related literature

For backgroud to tetra­zolate derivatives in coordination chemistry, see: Jiang *et al.* (2007 ▶); Song *et al.* (2009 ▶); Zhang (2009) ▶. For related structures, see: Facchetti *et al.* (2004 ▶); Lin *et al.* (2005 ▶); Vrbova *et al.* (2000 ▶)
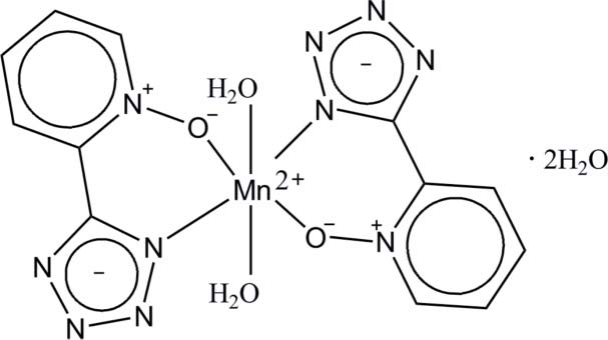


## Experimental

### 

#### Crystal data

[Mn(C_6_H_4_N_5_O)_2_(H_2_O)_2_]·2H_2_O
*M* *_r_* = 451.29
Monoclinic, 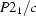

*a* = 6.4808 (13) Å
*b* = 12.034 (2) Å
*c* = 12.787 (4) Å
β = 116.24 (2)°
*V* = 894.5 (4) Å^3^
*Z* = 2
Mo *K*α radiation
μ = 0.80 mm^−1^
*T* = 293 K
0.10 × 0.10 × 0.08 mm

#### Data collection

Bruker SMART CCD area-detector diffractometer
Absorption correction: multi-scan (*SADABS*; Bruker, 2000 ▶) *T*_min_ = 0.925, *T*_max_ = 0.939
7432 measured reflections
1579 independent reflections
1102 reflections with *I* > 2σ(*I*)
*R*_int_ = 0.115

#### Refinement

*R*[*F* ^2^ > 2σ(*F* ^2^)] = 0.078
*wR*(*F* ^2^) = 0.133
*S* = 1.14
1579 reflections
133 parameters
H-atom parameters constrained
Δρ_max_ = 0.39 e Å^−3^
Δρ_min_ = −0.38 e Å^−3^

### 

Data collection: *SMART* (Bruker, 2000 ▶); cell refinement: *SAINT* (Bruker, 2000 ▶); data reduction: *SAINT*; program(s) used to solve structure: *SHELXS97* (Sheldrick, 2008 ▶); program(s) used to refine structure: *SHELXL97* (Sheldrick, 2008 ▶); molecular graphics: *SHELXTL* (Sheldrick, 2008 ▶); software used to prepare material for publication: *SHELXTL*.

## Supplementary Material

Crystal structure: contains datablocks global, I. DOI: 10.1107/S1600536811001620/lx2180sup1.cif

Structure factors: contains datablocks I. DOI: 10.1107/S1600536811001620/lx2180Isup2.hkl

Additional supplementary materials:  crystallographic information; 3D view; checkCIF report

## Figures and Tables

**Table 1:** Hydrogen-bond geometry (Å, °)

*D*—H⋯*A*	*D*—H	H⋯*A*	*D*⋯*A*	*D*—H⋯*A*
O3—H3*A*⋯N2	0.88	2.15	3.010 (5)	164
O2—H2*A*⋯O3^i^	0.84	2.01	2.756 (5)	147
O2—H2*B*⋯N3^ii^	0.86	2.06	2.858 (5)	154
O3—H3*B*⋯N4^ii^	0.82	2.10	2.917 (6)	176

Symmetry codes: (i) 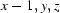; (ii) 
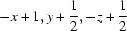
.

**Fig. 1.**
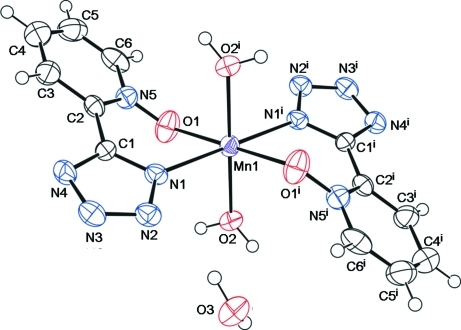
The molecular structure of the title compound with the atomic numbering scheme. Displacement ellipsoids are drawn at the 30% probability level. [Symmetry code: (i) - x + 1, - y + 1, - z + 1. ]

**Fig. 2.**
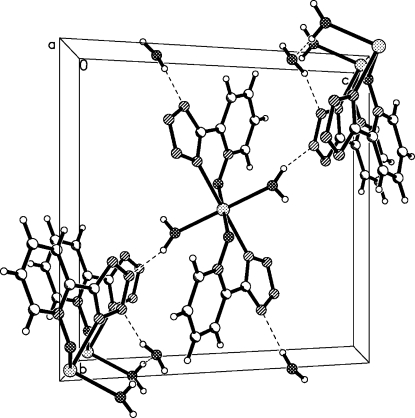
A view of the O–H···O and O–H···N hydrogen bonds (dotted lines) in the crystal structure of the title compound.

## supplementary crystallographic information

### Comment

Tetrazole as functional group plays an important role in coordination chemistry, medicinal chemistry and materials science applications (Song *et al.*, 2009; Jiang *et al.*, 2007; Zhang, 2009). It's interesting for the study of tetrazolate complexes to delineate the ways in which tetrazoles bind to metal centres. Here we report the structure of a novel substituted tetrazolato-metal complex, diaquabis(5-(2-pyridyl-1-oxide)tetrazolato)manganese(II) dihydrate.

The crystal structure of the title complex consists of the mononuclear manganese (II) unit [Mn(C_6_H_4_N_5_O)_2_(H_2_O)_2_], and two lattice water molecules (Fig. 1). In the mononuclear unit, manganese(II) ion is in a distorted octahedral environment, being six-coordinated by two N atoms and two O atoms from two bidentate 5-(2-pyridyl-1-oxide)tetrazolato-ligands, and two O atoms of two coordinated water molecules with Mn–O distances from 2.090 (4)Å to 2.209 (3) Å, Mn–N bond length of 2.255 (4)Å and O1–Mn1–N1 angle of 79.47 (14)°, which are comparable with the values observed in other metal-tetrazolate complexes (Vrbova *et al.*, 2000; Lin *et al.*, 2005; Facchetti *et al.*, 2004). The pyridine and tetrazole rings are twisted against each other by 20.466 (190)°. In the crystal structure, all the water H atoms are involved in O–H···N and O–H···O hydrogen bonds with the solvate water O (O3W) and the tetrazole N (N2, N4) atoms. The interactions link the molecules into a three dimensional network (Table 1 and Fig. 2).

### Experimental

A solution of 5-(2-pyridyl-1-oxide)tetrazole (32.6 mg, 0.2 mmol) and K_2_CO_3_ (13.8 mg, 0.1 mmol) in H_2_O (10 ml) was dropped slowly into a solution of Mn(ClO_4_).6H_2_O (36.2 mg, 0.1 mmol) dissolved in methanol (10 ml). The resulting brown suspension solution was stirred for 24 h at room temperature and filtered. Yellow crystals were separated from filtrate after about one month and collected for X-ray analysis (m.p. >573 K).

### Refinement

H atoms were placed in calculated positions, with C–H = 0.93Å and O–H = 0.82-0.88 Å, and included in the final cycles of refinement using a riding model, with *U*_iso_(H) = 1.2*U*_eq_(parent atom).

### Crystal data

**Table:**

[Mn(C_6_H_4_N_5_O)_2_(H_2_O)_2_]·2H_2_O	*F*(000) = 462
*M**_r_* = 451.29	*D*_x_ = 1.676 Mg m^−^^3^
Monoclinic, *P*2_1_/*c*	Mo *K*α radiation, λ = 0.71073 Å
Hall symbol: -P 2ybc	Cell parameters from 7005 reflections
*a* = 6.4808 (13) Å	θ = 3.1–27.6°
*b* = 12.034 (2) Å	µ = 0.80 mm^−^^1^
*c* = 12.787 (4) Å	*T* = 293 K
β = 116.24 (2)°	Block, yellow
*V* = 894.5 (4) Å^3^	0.10 × 0.10 × 0.08 mm
*Z* = 2	

### Data collection

**Table:**

Bruker SMART CCD area-detector diffractometer	1579 independent reflections
Radiation source: fine-focus sealed tube	1102 reflections with *I* > 2σ(*I*)
graphite	*R*_int_ = 0.115
Detector resolution: 10.0 pixels mm^-1^	θ_max_ = 25.0°, θ_min_ = 3.4°
φ and ω scans	*h* = −7→7
Absorption correction: multi-scan (*SADABS*; Bruker, 2000)	*k* = −14→14
*T*_min_ = 0.925, *T*_max_ = 0.939	*l* = −15→15
7432 measured reflections	

### Refinement

**Table:**

Refinement on *F*^2^	Primary atom site location: structure-invariant direct methods
Least-squares matrix: full	Secondary atom site location: difference Fourier map
*R*[*F*^2^ > 2σ(*F*^2^)] = 0.078	Hydrogen site location: difference Fourier map
*wR*(*F*^2^) = 0.133	H-atom parameters constrained
*S* = 1.14	*w* = 1/[σ^2^(*F*_o_^2^) + (0.0354*P*)^2^ + 1.2794*P*] where *P* = (*F*_o_^2^ + 2*F*_c_^2^)/3
1579 reflections	(Δ/σ)_max_ < 0.001
133 parameters	Δρ_max_ = 0.39 e Å^−^^3^
0 restraints	Δρ_min_ = −0.38 e Å^−^^3^

### Special details

**Table:**

Geometry. All e.s.d.'s (except the e.s.d. in the dihedral angle between two l.s. planes) are estimated using the full covariance matrix. The cell e.s.d.'s are taken into account individually in the estimation of e.s.d.'s in distances, angles and torsion angles; correlations between e.s.d.'s in cell parameters are only used when they are defined by crystal symmetry. An approximate (isotropic) treatment of cell e.s.d.'s is used for estimating e.s.d.'s involving l.s. planes.
Refinement. Refinement of *F*^2^ against ALL reflections. The weighted *R*-factor *wR* and goodness of fit *S* are based on *F*^2^, conventional *R*-factors *R* are based on *F*, with *F* set to zero for negative *F*^2^. The threshold expression of *F*^2^ > σ(*F*^2^) is used only for calculating *R*-factors(gt) *etc*. and is not relevant to the choice of reflections for refinement. *R*-factors based on *F*^2^ are statistically about twice as large as those based on *F*, and *R*- factors based on ALL data will be even larger.

### Fractional atomic coordinates and isotropic or equivalent isotropic displacement parameters (Å^2^)

**Table:**

	*x*	*y*	*z*	*U*_iso_*/*U*_eq_	
Mn1	0.5000	0.5000	0.5000	0.0282 (3)	
C1	0.4458 (9)	0.2469 (4)	0.4227 (4)	0.0260 (12)	
C2	0.3159 (8)	0.2280 (4)	0.4903 (4)	0.0276 (12)	
C3	0.2946 (10)	0.1223 (5)	0.5282 (5)	0.0406 (15)	
H3	0.3648	0.0631	0.5099	0.049*	
C4	0.1752 (11)	0.1017 (6)	0.5912 (5)	0.0531 (18)	
H4	0.1668	0.0303	0.6168	0.064*	
C5	0.0688 (10)	0.1883 (6)	0.6155 (5)	0.0508 (17)	
H2	−0.0165	0.1759	0.6568	0.061*	
C6	0.0860 (9)	0.2922 (5)	0.5800 (5)	0.0407 (15)	
H1	0.0140	0.3510	0.5979	0.049*	
N1	0.5349 (7)	0.3443 (3)	0.4111 (3)	0.0289 (10)	
N2	0.6423 (7)	0.3211 (4)	0.3443 (4)	0.0338 (11)	
N3	0.6179 (8)	0.2138 (4)	0.3188 (4)	0.0368 (12)	
N4	0.4943 (7)	0.1654 (4)	0.3670 (4)	0.0346 (11)	
N5	0.2085 (7)	0.3114 (4)	0.5179 (4)	0.0344 (11)	
O1	0.2069 (6)	0.4131 (3)	0.4819 (4)	0.0491 (11)	
O2	0.2698 (6)	0.5780 (3)	0.3322 (3)	0.0396 (10)	
H2A	0.1657	0.5331	0.2904	0.048*	
H2B	0.2775	0.6340	0.2925	0.048*	
O3	0.8363 (6)	0.4852 (3)	0.2336 (3)	0.0447 (10)	
H3A	0.7951	0.4450	0.2787	0.054*	
H3B	0.7388	0.5334	0.2038	0.054*	

### Atomic displacement parameters (Å^2^)

**Table:**

	*U*^11^	*U*^22^	*U*^33^	*U*^12^	*U*^13^	*U*^23^
Mn1	0.0324 (7)	0.0249 (6)	0.0313 (7)	−0.0007 (6)	0.0178 (5)	−0.0026 (6)
C1	0.028 (3)	0.024 (3)	0.025 (3)	0.001 (2)	0.011 (3)	0.002 (2)
C2	0.027 (3)	0.027 (3)	0.027 (3)	0.003 (2)	0.011 (3)	−0.005 (2)
C3	0.053 (4)	0.037 (4)	0.040 (4)	−0.001 (3)	0.028 (3)	−0.002 (3)
C4	0.069 (5)	0.050 (4)	0.046 (4)	−0.012 (4)	0.030 (4)	0.000 (3)
C5	0.046 (4)	0.069 (5)	0.038 (4)	−0.010 (4)	0.019 (3)	0.005 (4)
C6	0.033 (3)	0.061 (4)	0.035 (4)	0.005 (3)	0.021 (3)	−0.014 (3)
N1	0.030 (2)	0.033 (3)	0.026 (2)	0.000 (2)	0.016 (2)	−0.003 (2)
N2	0.032 (3)	0.041 (3)	0.032 (3)	−0.001 (2)	0.018 (2)	−0.003 (2)
N3	0.038 (3)	0.042 (3)	0.034 (3)	0.003 (2)	0.019 (2)	−0.007 (2)
N4	0.043 (3)	0.032 (3)	0.035 (3)	−0.002 (2)	0.024 (3)	−0.009 (2)
N5	0.035 (3)	0.029 (3)	0.034 (3)	0.004 (2)	0.011 (2)	0.002 (2)
O1	0.043 (3)	0.038 (2)	0.076 (3)	−0.0019 (19)	0.035 (2)	0.000 (2)
O2	0.049 (2)	0.038 (2)	0.030 (2)	−0.0062 (19)	0.016 (2)	0.0060 (18)
O3	0.046 (2)	0.040 (2)	0.055 (3)	0.005 (2)	0.028 (2)	0.012 (2)

### Geometric parameters (Å, °)

**Table:**

Mn1—O1	2.090 (4)	C4—H4	0.9300
Mn1—O1^i^	2.090 (4)	C5—C6	1.351 (8)
Mn1—O2	2.209 (3)	C5—H2	0.9300
Mn1—O2^i^	2.209 (3)	C6—N5	1.369 (6)
Mn1—N1	2.255 (4)	C6—H1	0.9300
Mn1—N1^i^	2.255 (4)	N1—N2	1.348 (5)
C1—N4	1.329 (6)	N2—N3	1.324 (6)
C1—N1	1.344 (6)	N3—N4	1.341 (6)
C1—C2	1.467 (7)	N5—O1	1.306 (5)
C2—N5	1.353 (6)	O2—H2A	0.8446
C2—C3	1.390 (7)	O2—H2B	0.8583
C3—C4	1.363 (7)	O3—H3A	0.8803
C3—H3	0.9300	O3—H3B	0.8172
C4—C5	1.359 (8)		
			
O1—Mn1—O1^i^	180.0	C5—C4—C3	118.3 (6)
O1—Mn1—O2	85.11 (15)	C5—C4—H4	120.9
O1^i^—Mn1—O2	94.89 (14)	C3—C4—H4	120.9
O1—Mn1—O2^i^	94.89 (15)	C6—C5—C4	120.4 (6)
O1^i^—Mn1—O2^i^	85.11 (14)	C6—C5—H2	119.8
O2—Mn1—O2^i^	180.000 (1)	C4—C5—H2	119.8
O1—Mn1—N1	79.47 (14)	C5—C6—N5	120.4 (5)
O1^i^—Mn1—N1	100.53 (14)	C5—C6—H1	119.8
O2—Mn1—N1	92.20 (14)	N5—C6—H1	119.8
O2^i^—Mn1—N1	87.80 (14)	C1—N1—N2	104.9 (4)
O1—Mn1—N1^i^	100.53 (14)	C1—N1—Mn1	121.7 (3)
O1^i^—Mn1—N1^i^	79.47 (14)	N2—N1—Mn1	133.4 (3)
O2—Mn1—N1^i^	87.80 (14)	N3—N2—N1	108.6 (4)
O2^i^—Mn1—N1^i^	92.20 (14)	N2—N3—N4	109.9 (4)
N1—Mn1—N1^i^	180.000 (1)	C1—N4—N3	104.9 (4)
N4—C1—N1	111.7 (4)	O1—N5—C2	121.8 (4)
N4—C1—C2	122.4 (4)	O1—N5—C6	116.5 (5)
N1—C1—C2	125.9 (4)	C2—N5—C6	121.6 (5)
N5—C2—C3	116.4 (5)	N5—O1—Mn1	124.4 (3)
N5—C2—C1	122.3 (5)	Mn1—O2—H2A	110.5
C3—C2—C1	121.2 (5)	Mn1—O2—H2B	135.8
C4—C3—C2	122.8 (6)	H2A—O2—H2B	111.6
C4—C3—H3	118.6	H3A—O3—H3B	107.3
C2—C3—H3	118.6		
			
N4—C1—C2—N5	−160.1 (5)	O2^i^—Mn1—N1—N2	−109.1 (4)
N1—C1—C2—N5	21.7 (8)	C1—N1—N2—N3	−0.5 (5)
N4—C1—C2—C3	19.1 (8)	Mn1—N1—N2—N3	177.0 (3)
N1—C1—C2—C3	−159.1 (5)	N1—N2—N3—N4	0.4 (5)
N5—C2—C3—C4	−0.7 (8)	N1—C1—N4—N3	−0.1 (6)
C1—C2—C3—C4	180.0 (5)	C2—C1—N4—N3	−178.5 (4)
C2—C3—C4—C5	1.4 (9)	N2—N3—N4—C1	−0.2 (5)
C3—C4—C5—C6	−1.4 (9)	C3—C2—N5—O1	−176.5 (5)
C4—C5—C6—N5	0.8 (9)	C1—C2—N5—O1	2.8 (7)
N4—C1—N1—N2	0.3 (6)	C3—C2—N5—C6	0.1 (7)
C2—C1—N1—N2	178.7 (5)	C1—C2—N5—C6	179.3 (5)
N4—C1—N1—Mn1	−177.5 (3)	C5—C6—N5—O1	176.6 (5)
C2—C1—N1—Mn1	0.9 (7)	C5—C6—N5—C2	−0.1 (8)
O1—Mn1—N1—C1	−27.4 (4)	C2—N5—O1—Mn1	−50.6 (6)
O1^i^—Mn1—N1—C1	152.6 (4)	C6—N5—O1—Mn1	132.7 (4)
O2—Mn1—N1—C1	−112.1 (4)	O2—Mn1—O1—N5	146.5 (4)
O2^i^—Mn1—N1—C1	67.9 (4)	O2^i^—Mn1—O1—N5	−33.5 (4)
O1—Mn1—N1—N2	155.5 (4)	N1—Mn1—O1—N5	53.3 (4)
O1^i^—Mn1—N1—N2	−24.5 (4)	N1^i^—Mn1—O1—N5	−126.7 (4)
O2—Mn1—N1—N2	70.9 (4)		

Symmetry codes: (i) −*x*+1, −*y*+1, −*z*+1.

### Hydrogen-bond geometry (Å, °)

**Table:**

*D*—H···*A*	*D*—H	H···*A*	*D*···*A*	*D*—H···*A*
O3—H3A···N2	0.88	2.15	3.010 (5)	164
O2—H2A···O3^ii^	0.84	2.01	2.756 (5)	147
O2—H2B···N3^iii^	0.86	2.06	2.858 (5)	154
O3—H3B···N4^iii^	0.82	2.10	2.917 (6)	176

Symmetry codes: (ii) *x*−1, *y*, *z*; (iii) −*x*+1, *y*+1/2, −*z*+1/2.
